# Two‐year efficacy of SNK01 plus pembrolizumab for non‐small cell lung cancer: Expanded observations from a phase I/IIa randomized controlled trial

**DOI:** 10.1111/1759-7714.14523

**Published:** 2022-06-06

**Authors:** Hyung Jun Park, Yong Man Kim, Jae Seob Jung, Wonjun Ji, Jae Cheol Lee, Chang‐Min Choi

**Affiliations:** ^1^ Department of Pulmonology and Critical Care Medicine, Asan Medical Center University of Ulsan College of Medicine Seoul South Korea; ^2^ Information Medicine Asan medical center Seoul South Korea; ^3^ NKMAX Co., Ltd. Seongnam South Korea; ^4^ University of Ulsan College of Medicine Seoul South Korea

**Keywords:** immunotherapy, nk cells, PD‐L1, pembrolizumab

## Abstract

**Background:**

A previous trial showed that autologous ex‐vivo expanded NK cell (SNK01) treatment combined with pembrolizumab showed better efficacy than pembrolizumab monotherapy in advanced non‐small cell lung cancer (NSCLC). This study was a 2‐year follow‐up of that previous study to determine the long‐term efficacy of the combination treatment.

**Methods:**

This trial included 20 patients with advanced NSCLC with a PD‐L1 tumor proportion score of 1% or greater who failed prior to front‐line platinum‐based therapy. The patients received pembrolizumab with low‐dose SNK01 (2 × 10^9^ cells/dose) or high‐dose SNK01 (4 × 10^9^ cells/dose), or pembrolizumab monotherapy. The primary study endpoint was overall survival (OS), and the secondary endpoint was progression‐free survival (PFS).

**Results:**

Two patients were excluded following serious adverse events. Among the 11 patients who died, five were from the NK groups (41.6%, *n* = 5/12), and six received pembrolizumab monotherapy (100%, *n* = 6/6). The estimated 2‐year survival rate was 58.3% versus 16.7% (pembrolizumab plus SNK01 vs. pembrolizumab monotherapy). The hazard ratio of pembrolizumab plus SNK01 compared with pembrolizumab monotherapy was 0.32 (95% CI: 0.1, 1.08, *p*‐value: 0.066). Although the median PFS was significantly higher in the pembrolizumab plus SNK01 group than in the pembrolizumab alone group, OS and PFS did not differ statistically between patients who received low doses of NK cells and those who received high doses of NK cells.

**Conclusions:**

Autologous NK cells can enhance the long‐term OS and PFS for NSCLC. A larger study is needed to confirm this result.

Clinical Research Information Service number: KCT0003463.

## Graphical abstract

This trial included 18 patients with advanced NSCLC with a PD‐L1 TPS >1% and randomized the patients to pembrolizumab with low‐dose SNK01 (2 × 109 cells/dose) or high‐dose SNK01 (4 × 109 cells/dose), or pembrolizumab monotherapy. The estimated 2‐year OS was 58.3% vs. 16.7% (pembrolizumab plus SNK01 vs. pembrolizumab monotherapy). The estimated 2 years PFS was 33.3% vs. 16.7% (HR: 0.69, 95% CI: 0.18–2.61) and OS was 66.7% vs. 50% (HR: 0.51 95% CI: 0.08–3.07) in high dose and low dose SNK01 group.

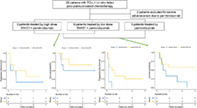

## INTRODUCTION

Lung cancer is the leading cause of cancer‐related death worldwide. Non‐small cell lung cancer (NSCLC) is the most common type of lung cancer, accounting for more than two‐thirds of cases.^1^ The identification of targetable gene alterations (i.e., EGFR, ALK, etc.) has increased the 5 year survival rate of lung cancer in Korea from 16.5% (in 2001–2005) to 30.2% (in 2013–2017),^2^ and incidence‐based mortality has also decreased from 35% for patients diagnosed in 2001 to 26% for patients diagnosed in 2014.^1^ This improved outcome can be explained by the introduction of new molecular‐targeted therapies and immune checkpoint inhibitors (i.e., drugs that target programmed cell death ligand 1 [PD‐L1]).

Pembrolizumab is a monoclonal antibody that binds the programmed cell death‐1 receptor (PD‐1) and blocks its interaction with PD‐L1 and PD‐L2.^3^ Because pembrolizumab acts on the PD‐L1 receptor, patients with a higher PD‐L1 tumor proportion score (TPS) show a better objective response rate and median overall survival (OS).^4^ The median progression‐free survival (PFS) and OS of patients treated with previous systemic therapy are 4 and 10.5 months, respectively.^3^, ^5^ Although pembrolizumab monotherapy showed better efficacy than conventional chemotherapy,^4^ other trials have been conducted to improve the efficacy of pembrolizumab monotherapy.

Natural killer (NK) cells are innate lymphocytes that have cytotoxic activity against cancer cells, which is mediated by the release of cytokines and chemokines.^6^ NK cells can recognize cancer cells either because the cancer cells lack ligands for inhibitory receptors expressed on NK cells, or because the cancer cell has a high expression of ligands that activate receptors found on NK cells.^7^ NK cell dysfunction is associated with advanced‐stage tumors in a mouse model of KRAS‐driven spontaneous lung cancer.^7^ In humans, NK cells isolated from NSCLC patients are fewer in number, have impaired IFN‐γ production and CD107a degranulation, and have lower cytotoxicity than controls.^7^ The CD56^bright^CD16^dim^ subset of NK cells are enriched in NSCLC tumor samples, although the role of this enriched subset is unclear. A recent randomized controlled trial (RCT) showed that infusion of NK cells in combination with pembrolizumab improved OS and PFS compared with pembrolizumab monotherapy in NSCLC patients, and multiple courses of NK cell infusion had a better outcome (OS 18.5 months) than a single NK cell infusion (OS 13.5 months).^6^

The initial randomized phase I/IIa clinical trial that forms the basis of the current extension study showed that the efficacy of pembrolizumab plus NK cells (SNK01) was better than pembrolizumab monotherapy; PFS for the combination was 6.2 months versus 1.6 months for pembrolizumab monotherapy.^8^

Although this result was consistent with that of the previous trial previous trial,^6^ the median follow‐up duration was only 17.5 months, which was not sufficient to evaluate the long‐term efficacy of pembrolizumab plus SNK01. The current study is an extension of the phase I/Iia clinical trial and evaluates the efficacy of pembrolizumab plus SNK01 therapy for more than 2 years to confirm the decreased hazardous ratio (HR) observed with the combination of SNK01 and pembrolizumab.

## METHODS

### Study design and patients

The randomized controlled trial design was previously reported.^8^ Patients with advanced or metastatic NSCLC (PD‐L1 TPS >1%) who had a history of previous platinum‐based therapy were included. All patients provided signed informed consent, and the study was conducted in full accordance with the Guidelines for Good Clinical Practice and the Declaration of Helsinki.

### Treatment and assessments

SNK01 is ex‐vivo expanded autologous NK cells. Peripheral blood mononuclear cells were filtered by leukapheresis and cultured by previous described method.^9^ The patients were randomized 1:1:1 to groups of pembrolizumab 200 mg only, pembrolizumab plus SNK01: 2 × 10^9^ cells/dose (low‐dose SNK01), or pembrolizumab plus SNK01: 4 × 10^9^ cells/dose (high‐dose SNK01).^8^ The groups who received pembrolizumab plus SNK01 were infused with SNK01 once a week for 6 weeks and with pembrolizumab (200 mg) once every 3 weeks. The pembrolizumab monotherapy group was treated with pembrolizumab (200 mg) once every 3 weeks.^8^

Tumor assessments were conducted by Response Evaluation Criteria in Solid Tumors criteria (RECIST v1.1), which were performed at baseline and then every 6 weeks until 52 weeks, and then every 12 weeks thereafter until progression of disease (PD) or any other symptoms that were considered to be a side‐effect of the drugs.^8^ After SNK01 treatment was discontinued in the NK cell groups, patients who were progression‐free were further treated with pembrolizumab.

Adverse events were assessed and graded by the Cancer Institute's Common Terminology Criteria for Adverse Event (CTCAE version 5.0). The causality for pembrolizumab and SNK01 assessed all the adverse events, and adverse events were categorized as an immune reaction when the adverse event was suspected to be related to pembrolizumab or SNK01.

### Outcomes

The primary endpoints were OS and PFS in the pembrolizumab plus SNK01 groups and the pembrolizumab monotherapy group within the intention‐to‐treat analysis. This study was initially evaluated as of July 15, 2020^8^; the current study is an analysis after 14 months of additional follow‐up (cutoff date of February 17, 2022). A subgroup analysis of OS and PFS was conducted in patients grouped by the number of NK cells/dose, the PD‐L1 TPS ratio, and their number of systemic anticancer treatments.

### Statistical analysis

The median follow‐up period was calculated using the reverse Kaplan–Meier method. OS and PFS were calculated by the Kaplan–Meier method. The HR was estimated with a Cox proportional‐hazards model. All statistical analyses were performed using R software version 4.0.5 (R Foundation for Statistical Computing).

## RESULTS

### Patient enrollment and follow‐up

Between February 2019 and March 2020, 20 patients were enrolled; 13 patients were male, and seven patients were female. Two patients were excluded due to side effects of pembrolizumab. Among the excluded patients, one patient in the pembrolizumab plus low‐dose SNK02 group showed arthralgia and myalgia (grade 3), and the other patient in the pembrolizumab plus high‐dose SNK01 group experienced pneumonia (grade 2). No patients from the pembrolizumab monotherapy group were excluded. Other side effects that occurred during the study period and the baseline characteristics are described in our previous report.^8^ At the time of data cutoff (February 17, 2022), the median follow‐up was 33 months, with a minimum follow‐up of 30 months. The baseline characteristics of the included patients are shown in Table 1.

**TABLE 1:** Baseline characteristics of included patients

Characteristics	Pembrolizumab monotherapy (*N* = 6)	SNK01 low dose (*N* = 6)	SNK01 high dose (*N* = 6)	*p*‐value
Age	58.0 ± 7.8	66.5 ± 5.5	57.7 ± 5.5	0.049
Sex				0.195
Male	2 (33.3%)	5 (83.3%)	4 (66.7%)	
Female	4 (66.7%)	1 (16.7%)	2 (33.3%)	
Smoking history				0.195
Non‐smoker	4 (66.7%)	1 (16.7%)	2 (33.3%)	
Ex‐smoker	2 (33.3%)	5 (83.3%)	4 (66.7%)	
Grade_2_AE				0.135
Absent	5 (83.3%)	2 (33.3%)	2 (33.3%)	
Present	1 (16.7%)	4 (66.7%)	4 (66.7%)	
EGFR				0.054
Negative	5 (83.3%)	2 (33.3%)	1 (16.7%)	
Positive	1 (16.7%)	4 (66.7%)	5 (83.3%)	
ALK				
Positive	6 (100.0%)	6 (100.0%)	6 (100.0%)	
PD‐L1 22c3 TPS				0.144
≧50	0 (0.0%)	2 (33.3%)	3 (50.0%)	
<50	6 (100.0%)	4 (66.7%)	3 (50.0%)	
Chemotherapy lines				0.014
≧3	6 (100.0%)	3 (50.0%)	1 (16.7%)	
2	0 (0.0%)	3 (50.0%)	5 (83.3%)	
Histology				0.347
Adenocarcinoma	6 (100.0%)	5 (83.3%)	6 (100.0%)	
Pleomorphic carcinoma	0 (0.0%)	1 (16.7%)	0 (0.0%)	

Abbreviation: TPS, tumor proportion score.

### Comparison between pembrolizumab plus SNK01 and pembrolizumab monotherapy

**FIGURE 1:**
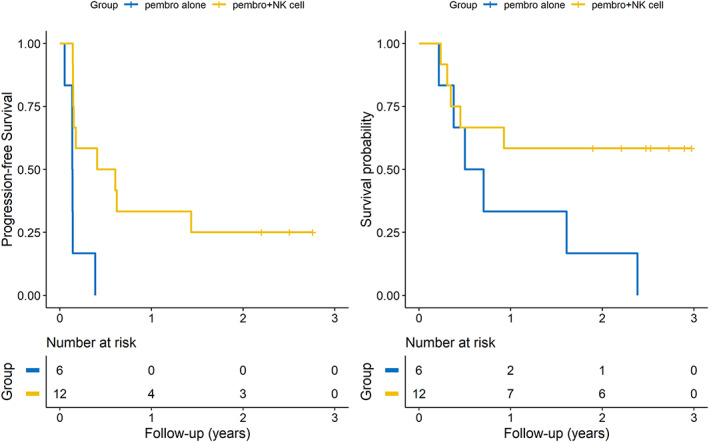
Kaplan–Meier curves of progression‐free survival (PFS) and overall survival (OS) for the pembrolizumab (pembro) plus SNK01 (NK cells) and pembrolizumab monotherapy groups

At the end of the observation period, 11 patients with the intention‐to‐treat population had died, including five patients from the pembrolizumab plus SNK01 groups (41.6%, *n* = 5/12) and six from the pembrolizumab monotherapy group (100%, *n* = 6/6). The estimated 2 year survival rate was 58.3% versus 16.7% (pembrolizumab plus SNK01 vs. pembrolizumab monotherapy). The HR of pembrolizumab plus SNK01 compared with pembrolizumab monotherapy for survival was 0.32 (95% CI: 0.1, 1.08, *p*‐value: 0.066). At the time of data cutoff, 15 of 18 patients had disease progression, including nine of 12 patients (75.0%) who were treated with pembrolizumab plus SNK01 and six of six patients (100%) who received pembrolizumab monotherapy. The estimated 2 year survival rates were 33.3% versus 16.7% (pembrolizumab plus SNK01 vs. pembrolizumab monotherapy). The median PFS was longer in the pembrolizumab plus SNK01 group than in the pembrolizumab monotherapy group, as reported in our previous study (Figure 1).

### Outcomes of the two doses of NK cells

**FIGURE 2:**
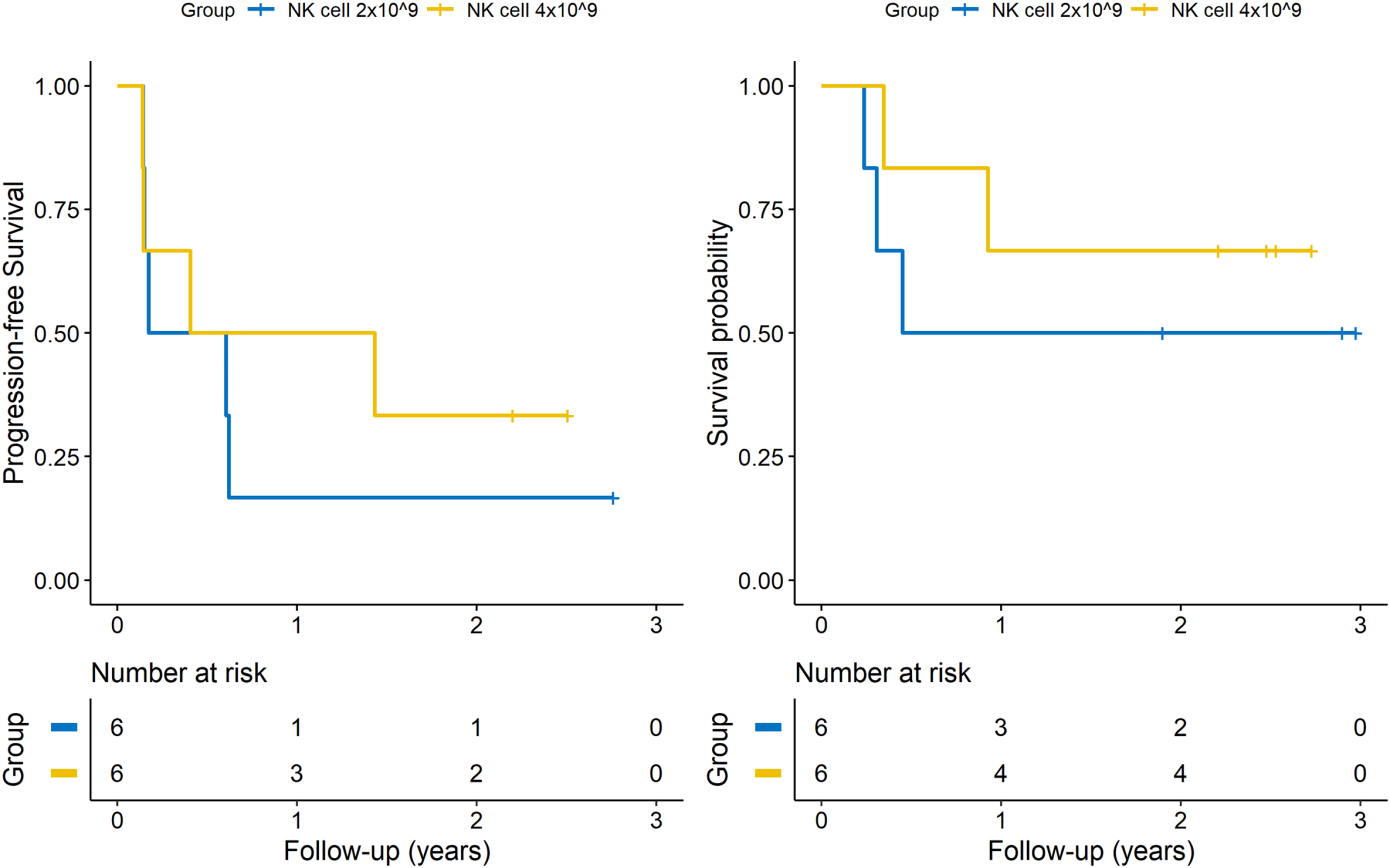
Kaplan–Meier curves of PFS and OS for pembrolizumab plus high‐dose and low‐dose SNK01 groups

Among the 12 patients treated with pembrolizumab plus SNK01, nine patients showed disease progression at the data cutoff date. The PFS and OS of the two NK groups are shown in Figure 2. The 2 year PFS rates were 16.7% in the low‐dose SNK01 group (2 × 10^9^ cells/dose) and 33.3% in the high‐dose SNK01 group (4 × 10^9^ cells/dose), and the HR estimated with PFS between the two dose groups was not statistically significant (HR: 0.68, 95% CI: 0.18–2.61, *p*‐value: 0.579). For OS, four of six patients (66.7%) in the low‐dose SNK01 and two of six patients (33.3%) in the high‐dose SNK01 group died. The estimated 2 year survival rates were 50% in the low‐dose SNK01 group and 66.7% in the high‐dose SNK01 group; the HR estimated with OS between the two dose groups was not statistically significant (HR: 0.51, 95% CI: 0.08–3.07, *p*‐value: 0.463).

### Subgroup analysis

**FIGURE 3:**
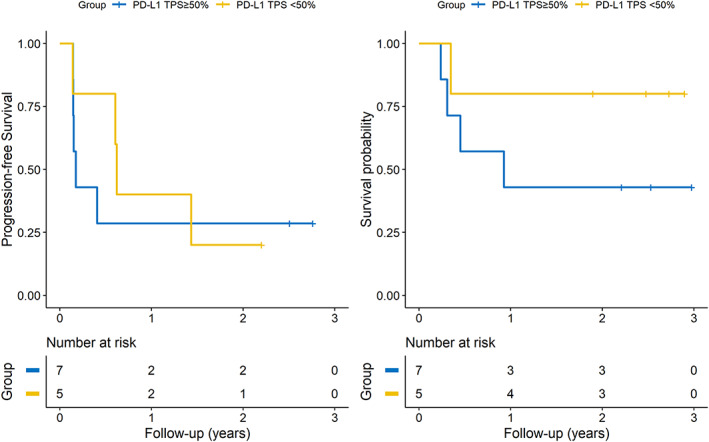
Kaplan–Meier curves of PFS and OS for high (>50%) and low (<50%) PD‐L1 tumor proportion score groups receiving pembrolizumab plus SNK01

Among the 12 patients treated with pembrolizumab plus SNK01, five patients had a PD‐L1 TPS of more than 50%, and seven patients had a PD‐L1 TPS of less than 50%. The estimated 2 year PFS was 20.0% versus 28.6% in patients with PD‐L1 < 50% and PD‐L1 ≥ 50%, respectively; the HR with PFS of the PD‐L1 TPS ≥50% group compared with the <50% TPS group was not statistically significant (HR 0.81, 95% CI: 0.21–3.08, *p*‐value: 0.754). The 2 year OS was 42.9% versus 80.0% in patients with PD‐L1 < 50% group and PD‐L1 > 50%, respectively; the HR with OS of the PD‐L1 TPS ≥50% group compared with the <50% TPS group was not statistically significant (HR 0.29, 95% CI: 0.03–2.59, *p*‐value: 0.267) (Figure 3).

**FIGURE 4:**
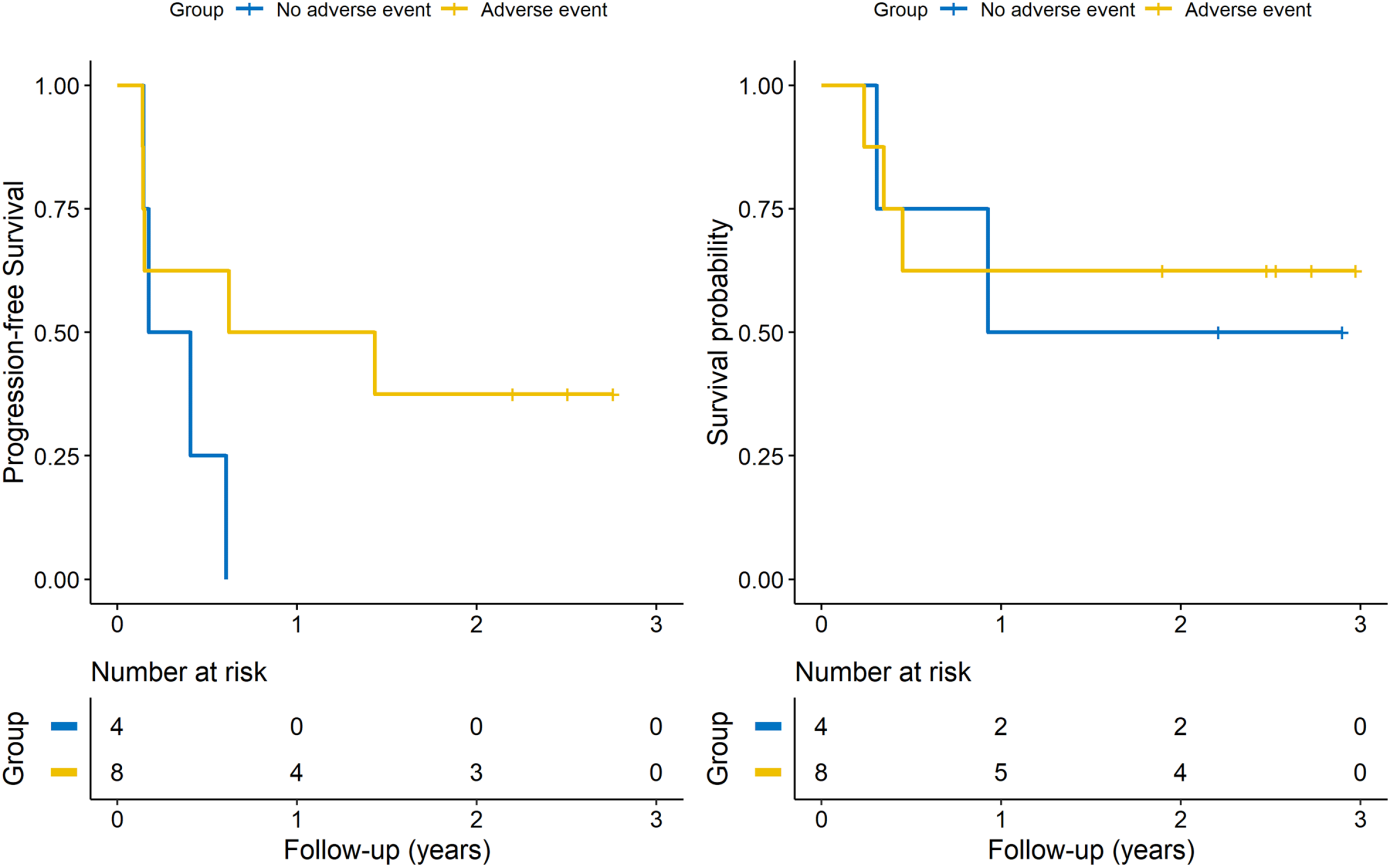
Kaplan–Meier curves of subgroups with or without immune‐related adverse effects in patients receiving pembrolizumab plus SNK01

Among the 12 patients treated with pembrolizumab plus SNK01, eight patients experienced immune‐related adverse events more severe than grade 2; four of six patients in the high‐dose SNK01 group, and four of six patients in the low‐dose SNK01 group. The median PFS was 12.3 months in the adverse event group and 3.5 months in patients who did not experience adverse events (HR 0.35, 95% CI: 0.08–1.59, *p*‐value: 0.172). The 2 year OS was 62.5% in the adverse event group and 50% in patients who did not experience adverse events (HR 0.78, 95% CI: 0.13–4.66, *p*‐value: 0.781) (Figure 4).

**FIGURE 5:**
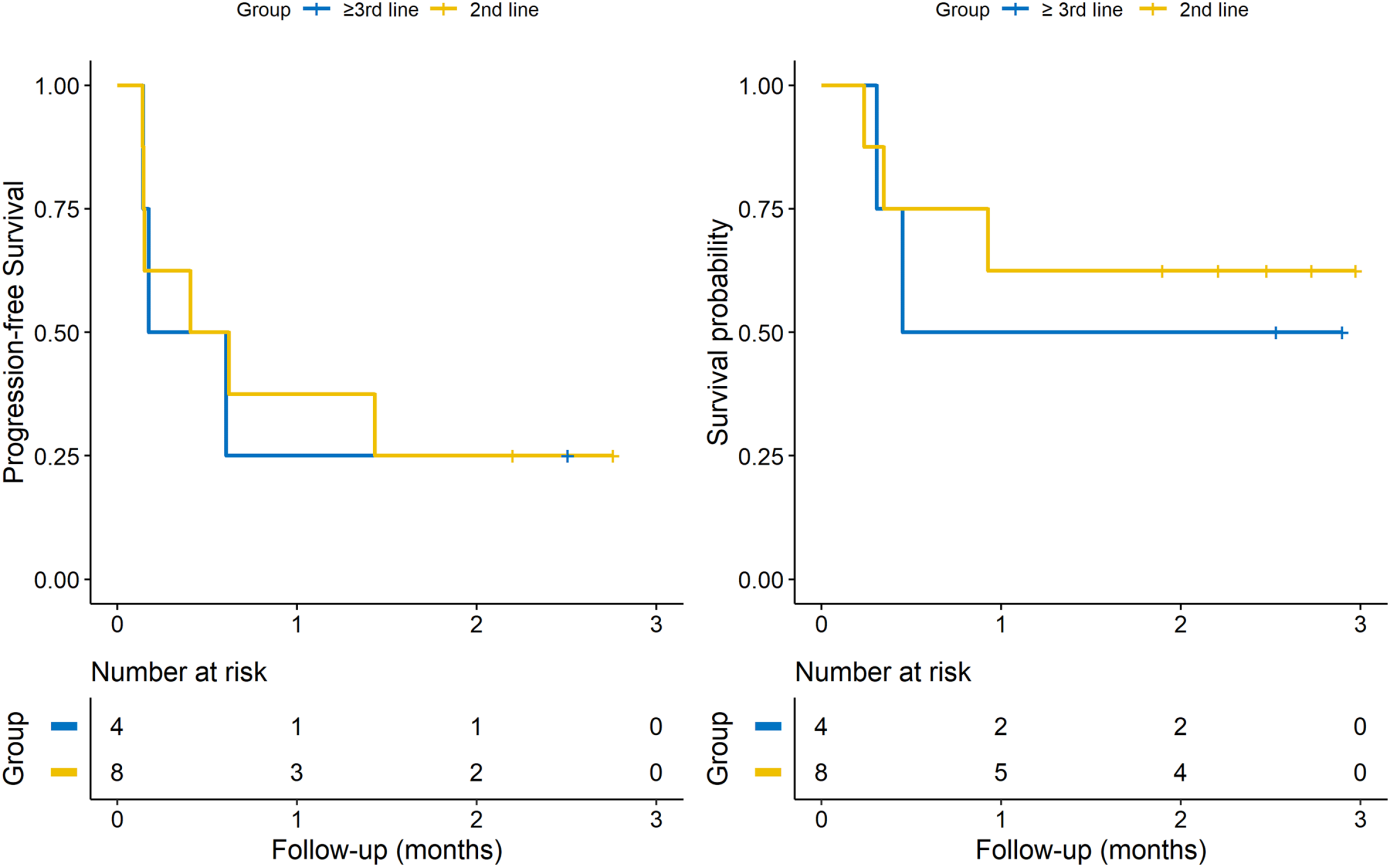
Kaplan–Meier curves of subgroups receiving SNK01 (plus pembrolizumab) as a second‐line treatment and a third‐line or higher treatment

Among the 12 patients treated with pembrolizumab plus SNK01 cells, four patients received SNK01 as a third‐line or higher systemic anticancer treatment, and eight patients received SNK01 as a second‐line treatment. In patients who received SNK01 as a second‐line treatment, the estimated 2 year PFS was 25%, and the OS was 50%, and in patients who received SNK01 as a third‐line or higher treatment, the PFS was 25%, and the OS was 62.5%. The HR of second‐line treatment compared with third‐line or higher treatment was not significant for PFS and OS (HR for PFS: 0.89, 95% CI: 0.22–3.59; HR for OS: 0.69, 95% CI: 0.11–4.17) (Figure 5).

## DISCUSSION

This study updated our previous RCT that showed pembrolizumab plus SNK01 had better efficacy than pembrolizumab monotherapy in NSCLC patients who had failed front‐line platinum‐based therapy. In the current study, the estimated 2 year OS and PFS for SNK01 groups were 58.3% and 33.3%, respectively. Although patient numbers in this study were small, we showed that the OS rate was higher in patients with PD‐L1 TPS ≥50% than in patients with PD‐L1 TPS <50%, and that was higher in patients received high‐dose SNK01 than low‐dose SNK01.

Pembrolizumab inhibits the PD‐1–PD‐L1 interaction, and this mechanism underlies its improved efficacy in NSCLC over conventional chemotherapy.^10^ Despite this mechanism, the median OS is only around 10 months when pembrolizumab is used as a second‐line treatment.^5^ The addition of NK cells to anticancer therapy may be a way to improve the efficacy of NSCLC treatments. NK cell dysfunction correlates with high tumor burden in mice, and the anticancer effect of NK cells is enhanced by the host's immunological response, therefore enhancing NK cell function has been highlighted as a therapeutic strategy in NSCLC.^11^ The function of NK cells in a patient can be enhanced in several ways, including cytokine therapy (IL‐2 treatment), monoclonal antibody treatment (cetuximab, pembrolizumab), adoptive transfer of activated NK cells (autologous and allogeneic), and genetic modification of NK cells.^11^ Pembrolizumab, which blocks PD‐1 activity, could enhance NK cell activity and the cytotoxic properties of NK cells.^12^ A previous RCT showed that the addition of allogeneic NK cells to pembrolizumab therapy enhanced the anticancer efficacy of pembrolizumab; OS and PFS were improved.^6^ In addition, many other clinical trials are evaluating the efficacy of autologous and allogeneic NK cells for NSCLC and small‐cell lung cancer.^11^ Our study showed that autologous NK cells improved the efficacy of pembrolizumab for treating NSCLC.

Regarding the dose of NK cells, our data showed that high‐dose NK cells tended to be more efficacious in OS and PFS than low‐dose NK cells. In a previous RCT involving allogeneic NK cells, multiple infusions of NK cells improved PFS and OS compared with a single NK infusion.^6^ Considering that our data support the previous observation of NK cell efficacy, the efficacy of NK cells could be dose‐dependent, although there were differences in the dosing (particularly the high dose) and the number of infusions between the current study and the previous study.^6^ It is well‐known that a higher PD‐L1 TPS is a positive prognostic factor for NSCLC patients treated with pembrolizumab.^13^ The survival benefit of allogeneic NK cells in a subgroup population who have PD‐L1 TPS ≥50% was previously demonstrated.^6^ Our data show that the survival benefit of NK cells was more dominant in patients with a PD‐L1 TPS ≥50% than in patents with a TPS <50%, although the statistical significance was not demonstrated due to the small number of participants. Considering that the estimated 2 year OS of pembrolizumab plus SNK01 in patients with PD‐L1 TPS >50% was 80%, our results suggest that combining pembrolizumab with NK cell infusion could benefit previously treated NSCLC patients.

The immune‐related side effects observed in this study and our previous phase study tended to be more prevalent in the NK cell groups,^8^ which might be related to the increased efficacy of pembrolizumab plus SNK01 compared with pembrolizumab monotherapy.^14^ However, this observation was not consistent with a previous RCT of allogeneic NK cells that showed no difference in adverse events between the NK cell group and the pembrolizumab monotherapy group.^6^ Thus, the true occurrence of immune‐related side effects should be further analyzed in future research. The occurrence of immune‐related adverse events in response to an immune checkpoint inhibitor could be used as a positive prognostic factor.^15^ In the current study, there was no difference in the prevalence of immune‐related adverse reactions between the high‐ and low‐dose SNK01 groups. The efficacy of NK cells with pembrolizumab (in respect to PFS but not OS) was higher in the patients who experienced adverse events. The three patients who experienced adverse events did not have disease progression at the data cutoff, and their disease was controlled with follow‐up pembrolizumab therapy. This observation should be evaluated in a future study that assesses whether immunological adverse reactions are a positive prognostic factor for the efficacy of the adoptive transfer of activated NK cells.

There are some limitations to this study. As this study was designed as a phase I/IIa RCT, there were not enough patients to see statistical significance in the subgroup analysis of efficacy. However, our results were similar to those of the RCT that used allogeneic NK cells; a further large‐scale study is needed to confirm if the results of the current study are statistically significant. Second, the design of the current study was different to a previous study^6^ with respect to doses and the types of NK cells used (autologous vs. allogeneic). Thus, when comparing the results of studies that use NK cells, differences in the NK cell regimen should be considered. Despite the different study design, our results are consistent with previously reported results,^6^ and the efficacy of NK cells could therefore be interpreted as an effect that is common to different types of NK cell therapy. Third, the baseline characteristics between the NK cell groups and the pembrolizumab monotherapy group were different. The PD‐L1 TPS of the pembrolizumab monotherapy group was less than 20%, which could exacerbate the efficacy of NK cells. To estimate the efficacy in patient groups with similar TPS, we compared the pembrolizumab monotherapy group to the patient subgroup receiving pembrolizumab plus SNK01 groups that had a PD‐L1 TPS <20%. Although statistical differences were shown only on PFS (not on OS), the OS of the subgroup receiving pembrolizumab plus SNK01 was more prolonged than in the pembrolizumab monotherapy group (Figure S1).

In conclusion, this study compared the long‐term efficacy of pembrolizumab plus autologous ex‐vivo expanded NK cells (SNK01) with pembrolizumab monotherapy. The results showed that autologous NK cells enhance the long‐term OS and PFS in patients with NSCLC. A larger study is needed to confirm this result.

## CONFLICT OF INTEREST

All authors declare that there is no conflict of interest.

## Supporting information


**Figure S1** Kaplan–Meier curves for patients with PD‐L1 TPS <20% treated with NK cells and pembrolizumab (pembro) or pembrolizumab monotherapy
