# Supplementary material for: Two‐year efficacy of SNK01 plus pembrolizumab for non‐small cell lung cancer: Expanded observations from a phase I/IIa randomized controlled trial
Source: Thorac Cancer. 2022 Jun 6;13(14):2050–6. doi: 10.1111/1759-7714.14523 (PMC9284127; doi:10.1111/1759-7714.14523)

Supplemental Figure 1. Kaplan-Meier curves for patients with PD-L1 TPS <20% treated with NK cells and pembrolizumab (pembro) or pembrolizumab monotherapy


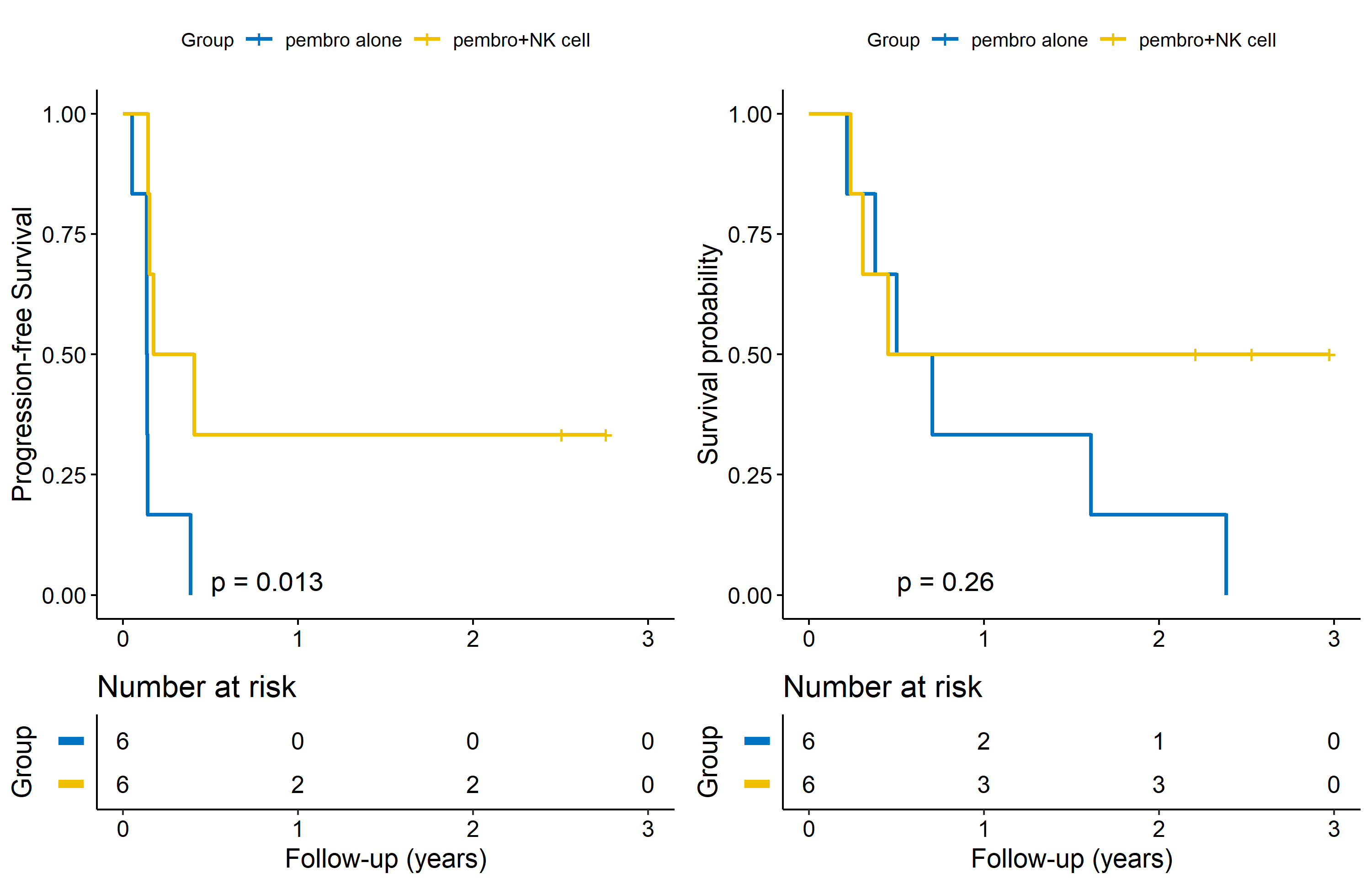

Supplement: Supplementary file 1 — Figure S1 Kaplan–Meier curves for patients with PD‐L1 TPS <20% treated with NK cells and pembrolizumab (pembro) or pembrolizumab monotherapy [file TCA-13-2050-s001.docx]
